# Facilitation of mouse skin-derived precursor growth and yield by optimizing plating density

**DOI:** 10.1515/biol-2021-0128

**Published:** 2021-12-10

**Authors:** Yiming Li, Lidan Xiong, Jie Tang, Ru Dai, Shiyi Li, Li Li

**Affiliations:** Department of Dermatology and Venerology, West China Hospital, Sichuan University, 37 Guoxue Alley, Chengdu, Sichuan Province 610041, China; Laboratory of Dermatology, Clinical Institute of Inflammation and Immunology (CIII), Frontiers Science Center for Disease-related Molecular Network, West China Hospital, Sichuan University, 37 Guoxue Alley, Wuhou District, Chengdu, Sichuan Province 610041, China; Department of Dermatology, 2nd Affiliated Hospital, School of Medicine, Zhejiang University, Hangzhou, Zhejiang Province 310009, China; Laboratory of Ethnopharmacology, West China Hospital, Sichuan University, Gaopeng Avenue, Gaoxin District, Chengdu, Sichuan Province 610041, China

**Keywords:** skin-derived precursors, dermal mesenchymal stem cells, density, cell culture, induced differentiation

## Abstract

Multiple methodologies have been reported to facilitate skin-derived precursor (SKP) growth, but the impact of plating density on SKP growth has not been studied. To determine the optimal plating density, we used six plating densities and two types of flasks for mouse SKP (mSKP) culture. On the 14th day, the number, diameter, and viability of mSKP spheres were compared by morphological assessment and cell counting kit 8, and we found the optimal plating density was 2.5 × 10^5^–5 × 10^5^ cells/mL. In addition, we investigated the correlation between the SKP spheres and the adherent cell colonies in the serum-free culture system. We treated the adherent cell colonies with two culture conditions and characterized the cells generated from two conditions by immunocytochemistry and induced differentiation, respectively. The results elucidated that the adherent cell colonies differentiated into either mSKPs or dermal mesenchymal stem cells under appropriate culture conditions. In conclusion, mSKP spheres differentiated from the adherent cell colonies. The optimal plating density significantly promoted and advanced the proliferation of adherent cell colonies, which optimized mSKP growth and yield. The adherent cell colonies possessed the capacity of differentiating into different types of cells under appropriate culture conditions.

## 1 Introduction

Persistent within the dermis throughout adulthood, skin-derived precursors (SKPs) are multipotent dermal stem cells that share multiple properties with embryonic neural crest stem cells [1,2]. SKPs are deemed to be promising in regenerative medicine and cell replacement therapy, given their capacity of the neuron [3], Schwann cell- [4], insulin-producing cell- [5], corneal epithelial-like cell- [6], skeletogenic cell- [7], and mesenchymal cell [8]-induced differentiation. A recent study demonstrated that except for the difference in morphology, stem cell antigen expression, and cell cycle distribution, mouse SKPs (mSKPs), and dermal mesenchymal stem cells (DMSCs) possessed distinct transcriptome profiles. Significantly enriched immune or inflammation-related differentially expressed genes and Kyoto encyclopedia of genes and genomes pathways presented in mSKPs [9]. These findings implied mSKPs’ possible application in immune modulation, which was in accordance with the preceding research [10].

Traditionally, SKPs were isolated according to the standard protocol described by Biernaskie et al. [11], but the growth and production remained limited. Multiple methodologies to promote SKP proliferation and sphere formation have been reported, including optimizing growth factor concentration [12], introducing transforming growth factor-β (TGF-β) [13], or fibroblast growth factor binding protein (FGF-BP) [14], employing stirred suspension bioreactors [15], and applying hydrogel scaffolds [16]. Some researchers even achieved induction of SKP-like spheres from established dermal cultures or primary fibroblast cultures [17,18,19].

The efficacy of the methodologies mentioned above varies. For instance, 1 ng/mL TGF-β increased the number of mSKP spheres by 2.6 times and increased the diameter by 1.5 times [13]. The culture medium containing 10 ng/mL FGF-BP and 40 ng/mL bFGF increased the number of mSKP spheres by 1.8 times [14]. The 60 rpm stirred suspension bioreactors raised the density of human SKPs (hSKPs) 4–5 times [15]. One major concern is these methodologies are apparatus-demanding or expensive and may alter SKP’s biological characteristics. Expensive and challenging cell culture and yet unsatisfactory production have hampered further research and clinical application. Cell growth optimization would be vital.

The growth rate of SKPs is cell density dependent, so that the lower the cell density, the slower their growth [11]. To date, no detailed research concerning the impact of plating density has been reported. In addition, no study on the adherent cell colonies in the serum-free SKP culture system was performed. In this study, we monitored the impact of various plating densities on mSKP proliferation without altering other culture conditions. We then explored the biological characteristics of adherent cell colonies. This study optimized mSKP growth and accelerated further research and possible clinical application.

## 2 Materials and methods

### 2.1 Animals and ethical approval

Cell suspension was collected from neonatal male Balb/C mice (aged 1–3 days) dermis according to the standard culture protocols [11] and conducted routinely in our lab [9,20,21,22,23].


**Ethical approval:** The research related to animal use has been complied with all the relevant national regulations and institutional policies for the care and use of animals and was approved by the Animal Ethics Committee of West China Hospital, Sichuan University (Approval No. 2017064A).

### 2.2 Culture medium setup

The SKP culture medium used in the study was commercially available DMEM/F12 (3:1, Invitrogen, USA), containing 0.1% penicillin/streptomycin (Invitrogen, USA), 40 ng/mL bFGF (Millipore, USA), 20 ng/mL EGF (Millipore, USA), and 2% B27 supplement (Gibco, USA). The culture medium for DMSC was low glucose DMEM (Invitrogen, USA), containing 10% FBS (Clarks, Australia) and 1% penicillin/streptomycin. The basal medium for spheres was SKP culture medium containing 5% FBS. The basal medium for adherent cell colonies was the same as the DMSC culture medium.

### 2.3 mSKP cell isolation

Neonatal male Balb/C mice dorsal skin was dissected and cut into 2–3 mm^2^ pieces. These dissected pieces were washed 3 times with PBS (Solarbio, Beijing, China) and digested with 0.1% trypsin (Invitrogen, USA) under gentle agitation for 1 h at 37°C. When tissue pieces became pale, they were washed 3 times with PBS. The epidermis was then removed from the dermis. Afterward, dermis pieces were digested by collagenase type XI (Sigma-Aldrich, USA) for 1 h at 37°C, mechanically dissociated with scissors, and subsequently triturated repeatedly in SKP culture medium with a 1,000 µL pipette tip. The supernatant was collected, and the trituration was repeated until tissue pieces became thin. After the dissociated cell suspension was filtered through a 40 µm cell strainer and centrifuged at 1,200 rpm for 7 min, the pellet was suspended in SKP culture medium.

### 2.4 mSKP culture at six plating densities and in two types of culture flasks

**Figure 1:**
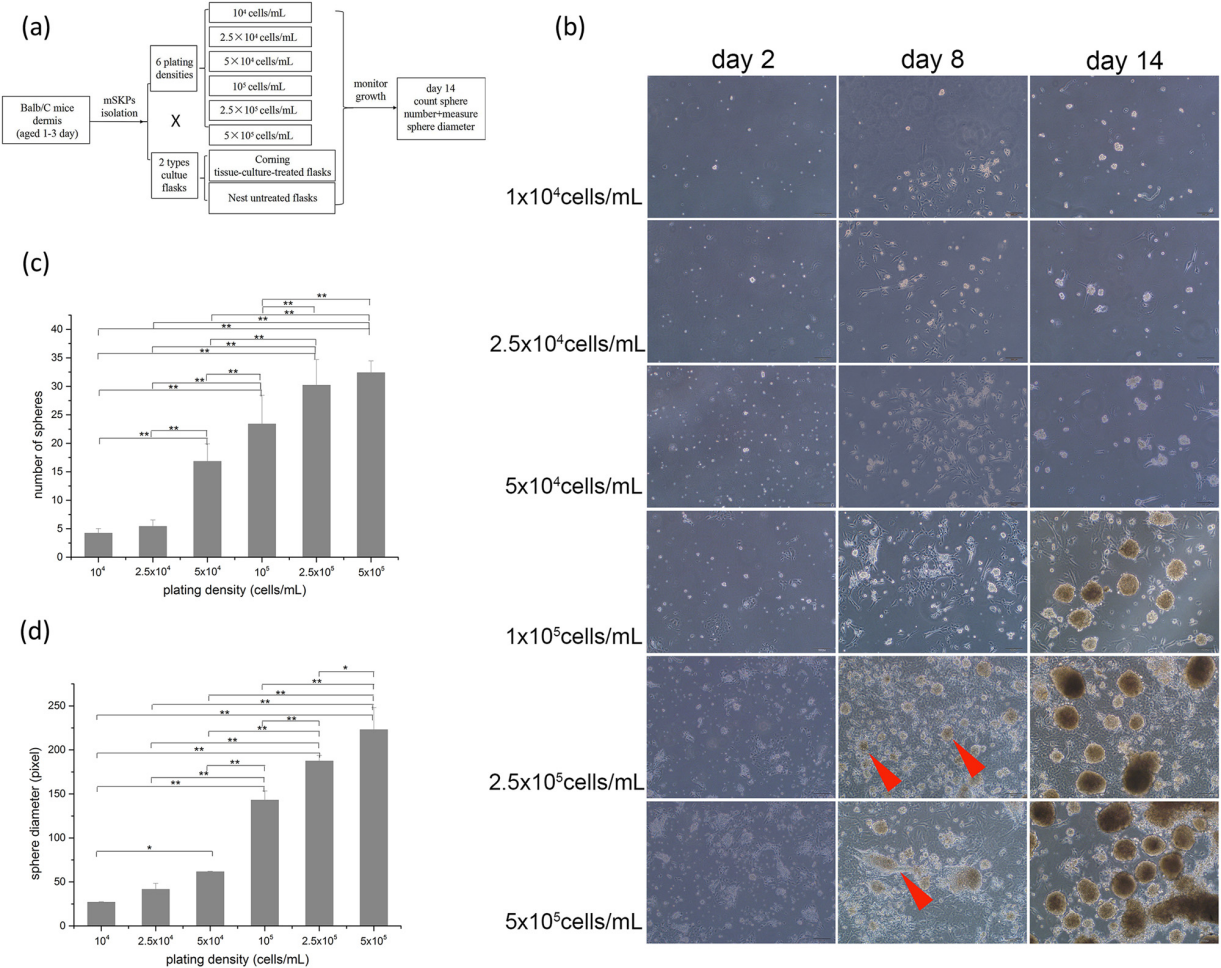
The experiment design for determining the optimal plating density and mSKP growth at multiple plating densities in Corning tissue-culture-treated flasks. (a) The experiment design; (b) mSKP growth at multiple plating densities in Corning tissue-culture-treated flasks. Adherent cell colonies are indicated by arrowheads; (c) diagram representing the number of mSKP spheres on the 14th day; (d) diagram representing the diameter of mSKP spheres on the 14th day. Bar: 100 µm. (Results of three independent experiments, ANOVA for multiple comparisons, *p < 0.05, **p < 0.01.).

After achieving the cell suspension mentioned in the “mSKP cell isolation” part, we detected the cell density by hemocytometer (Millipore, USA). Six plating densities (10^4^, 2.5 × 10^4^, 5 × 10^4^, 10^5^, 2.5 × 10^5^, 5 × 10^5^ cells/mL) were used. Meanwhile, two types of flasks, including Corning tissue-culture-treated flasks (Corning, NY, USA) and Nest untreated flasks (Nest, Wuxi, China), were used for mSKP culture. The final volume was 5 mL in a 25 cm^2^ flask. Cultures were fed every 3 days with an addition of 1 mL fresh medium containing all growth factors and supplements (bFGF, EGF, B27) at a concentration that would replenish the entirety of the culture medium. The proliferation of mSKPs at different plating densities in different flasks was monitored and recorded using a light microscope (Olympus, Japan) daily. The experiment design for this section can be found in Figure 1a.

### 2.5 mSKP sphere counting and diameter measurement

On the 14th day, five pictures were taken randomly for every culture condition, mSKP spheres were counted, and their diameters were measured using Image-pro Plus 7.0 (Media Cybernetics, USA). Then the number and diameter of mSKP spheres obtained from every culture condition were compared.

### 2.6 Cell counting kit 8 (CCK8) viability assay

The mSKP viability under every culture condition was tested using CCK8 (Dojindo, Japan) according to the manufacturer’s instructions. Briefly, CCK8 detected cell viability based upon intracellular WST-8 (C_20_H_14_N_6_NaO_11_S_2_) concentration, a compound similar to methyl thiazolyl tetrazolium. WST-8 could be transformed to orange-colored formazan through a reduction reaction catalyzed by dehydrogenases located in mitochondria. The faster the cell proliferate, the darker the orange color would be. The color shade and the number of cells present a linear relation for the same cell line.

### 2.7 Treat adherent cell colonies with culture condition A or B

mSKPs were cultured in Corning tissue-culture-treated flasks at the plating density of 5 × 10^5^ cells/mL. On the 10th day, both spheres and adherent cell colonies were present. The spheres were then poured out and transferred to the new flasks.

After being washed with PBS 5 times, the original flasks containing the adherent cell colonies were divided into groups A (culture condition A) and B (culture condition B), with 5 flasks in each group. Around 5 mL SKP culture medium was added to group A flasks, and 5 mL DMSC culture medium was added to group B flasks. For group A, cultures were fed every 3 days with an addition of 1 mL fresh medium containing all growth factors and supplements at a concentration that would replenish the entirety of the culture medium. For group B, we changed the fresh DMSC culture medium every 3 days. Cell growth was monitored, and pictures were taken daily. On the 8th day, the cell colonies generated from both groups were collected for characterization by immunocytochemistry and induced differentiation, respectively. The poured-out spheres were cultured with SKP medium in new flasks and fed every 3 days with a fresh medium containing all growth factors and supplements. The experiment design for this section can be found in Figure 4a.

### 2.8 Immunocytochemistry assay for cell colonies generated from culture condition A or B

Cells were plated on slides, fixed by 4% paraformaldehyde. The fixed cells were blocked with 3% BSA for 30 min and subsequently incubated with primary antibody overnight at 4°C. After being washed with PBS 3 times, cells were incubated with a secondary antibody for 1 h at room temperature. Finally, cells were incubated with DAPI (Dogindo, Japan) for 1 min. Primary antibodies were anti-α-SMA (Abcam, UK, 1:500), anti-Nanog (Abcam, UK, 1:250), anti-Oct4 (Abcam, UK, 1:250), anti-Pan Cytokeratin (Boster, Wuhan, 1:1,000), anti-Ssea 4 (Abcam, UK, 1:250), anti-Versican (Boster, Wuhan, 1:250), anti-Vwf (Boster, Wuhan, 1:500), anti-Fibronectin (Abcam, UK, 1:250), anti-Vimentin (Abcam, UK, 1:200), anti-Nestin (Abcam, UK, 1:500), anti-Sox2 (Boster, Wuhan, 1:250), and anti-Collagen I (Abcam, UK, 1:500). Secondary antibodies were Alexa Fluor^®^ 488 donkey anti-mouse (Abcam, UK, 1:500) and Alexa Fluor^®^ 594 goat anti-rabbit (Abcam, UK, 1:500). The protocol was performed in triplicate for both cell types.

### 2.9 Osteocyte-, adipocyte-, and chondrocyte-induced differentiation for cell colonies generated from culture condition A or B

Cells were trypsinized, dissociated into single cells, and then resuspended and cultured in basal medium for spheres or adherent cell colonies. The basal medium was replaced by osteocytes or adipocytes differentiation medium (Cyagen, USA) when the confluency reached 60–70%. The following steps were conducted according to the protocol within the kits. At the end of a 28 day induced differentiation, cells were stained with Alizarin Red Solution or Oil Red Solution provided in the kits, respectively.

For chondrocyte-induced differentiation, cells were collected, induced, and differentiated according to the protocol within the chondrogenic differentiation kit (Cyagen, USA). The chondrogenic pellets were harvested after 30 days in the 15 mL tubes culture. According to the protocol, pellets were 10% formalin-fixed, paraffin-embedded, machine-sliced, and then stained with Alcian blue (Leagene biotechnology, Beijing).

### 2.10 Statistical analysis

All data were expressed as mean value ± SE. Statistical significance was evaluated by analysis of variance (ANOVA) for multiple comparisons. A value of *p* < 0.05 was considered statistically significant.

## 3 Results

### 3.1 mSKP growth in Corning tissue-culture-treated flasks

In Corning tissue-culture-treated flasks, compared with lower plating densities (10^4^–10^5^ cells/mL), the plating densities of 2.5 × 10^5^ and 5 × 10^5^ cells/mL significantly promoted mSKP proliferation (*p* < 0.01). A large number of adherent cell colonies were present on the 5th or 6th day (Figure 1b, indicated by arrowheads). mSKPs then started to form on the adherent cell colonies on the 7th or 8th day. They were attached to the adherent cell colonies and then detached and migrated out to form floating mSKP spheres as they grew. On the 14th day, the number and the diameter of mSKP spheres generated from different plating densities varied significantly (*p* < 0.01) (Figure 1b–d). Compared with 10^4^ cells/mL, the number of mSKP spheres increased by 7.7 times, and the diameter of mSKP spheres increased by 8.3 times at the plating density of 5 × 10^5^ cells/mL (Figure 1c and d and Table 1).

**Table 1:** Number and diameter of mSKP spheres under multiple plating densities

Plating density (cells/mL)	Corning tissue-culture-treated flasks (mean ± SE)		Nest untreated flasks (mean ± SE)	
	Number of mSKP spheres	Diameter of mSKP spheres (pixel)	Number of mSKP spheres	Diameter of mSKP spheres (pixel)
10^4^	4.2 ± 0.84	26.96 ± 0.66	5.6 ± 1.14	24.58 ± 3.62
2.5 × 10^4^	5.4 ± 1.14	41.50 ± 6.91	7.8 ± 1.30	46.62 ± 0.52
5 × 10^4^	16.8 ± 3.11	61.42 ± 0.66	12.2 ± 2.59	72.43 ± 3.47
10^5^	23.4 ± 5.03	142.93 ± 10.50	30.2 ± 4.15	116.39 ± 15.40
2.5 × 10^5^	30.2 ± 4.49	187.37 ± 6.30	32.0 ± 3.54	182.80 ± 1.10
5 × 10^5^	32.4 ± 2.07	222.99 ± 25.20	33.8 ± 5.07	230.81 ± 10.91

### 3.2 mSKP growth in Nest untreated flasks

**Figure 2:**
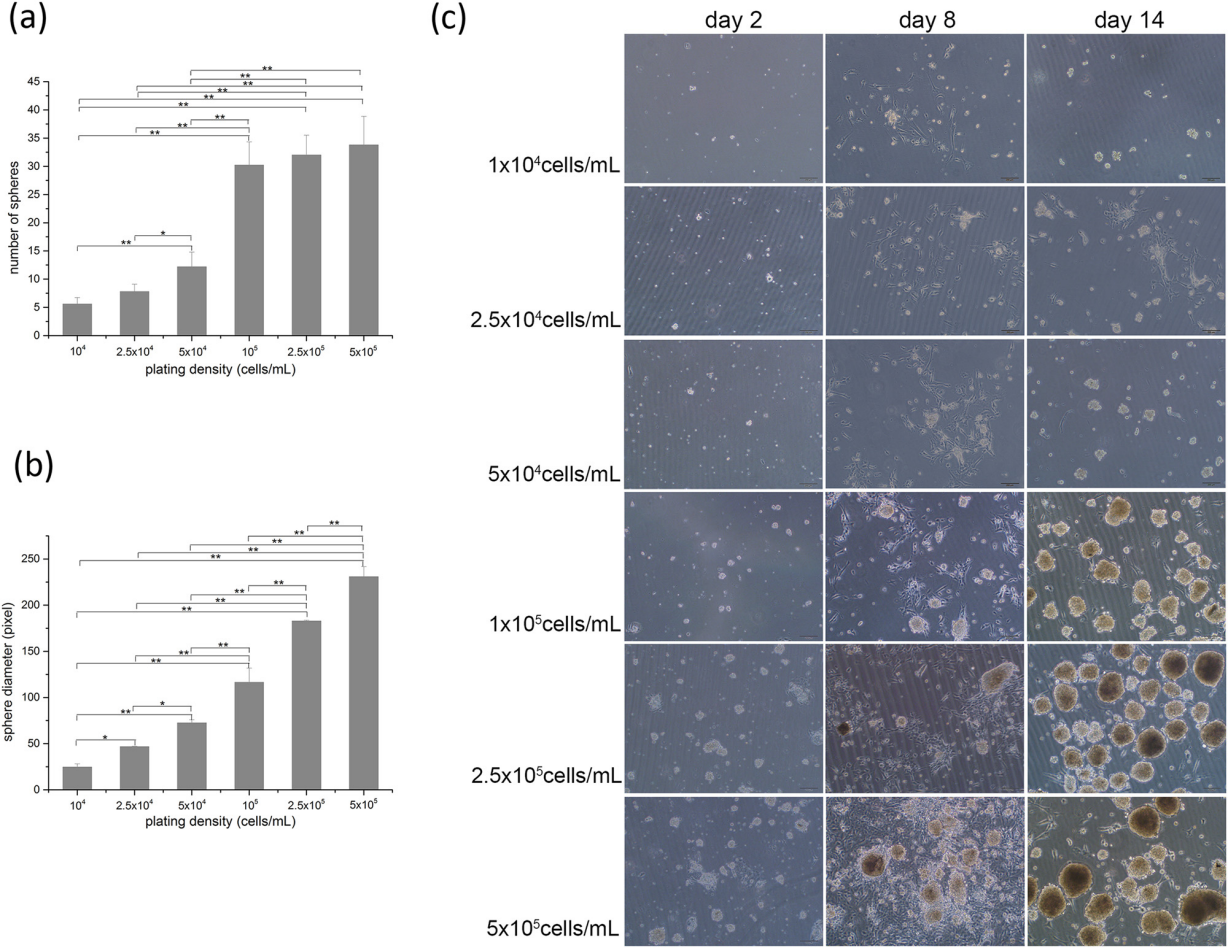
mSKP growth at multiple plating densities in Nest untreated flasks. (a) Diagram representing the number of mSKP spheres on the 14th day; (b) diagram representing the diameter of mSKP spheres on the 14th day; (c) mSKP growth at multiple plating densities in Nest untreated flasks. Bar: 100 µm. (Results of three independent experiments, ANOVA for multiple comparisons, *p < 0.05, **p < 0.01.).

A similar phenomenon was present in Nest untreated flasks (Figure 2). On the 14th day, the number and the diameter of mSKP spheres generated from different plating densities varied significantly (*p* < 0.01) (Figure 2a–c). Compared with 10^4^ cells/mL, the number of mSKP spheres increased by 6 times, and the diameter of mSKP spheres increased by 9.4 times when the plating density was 5 × 10^5^ cells/mL (Figure 2a and b and Table 1).

### 3.3 mSKP viability

**Figure 3:**
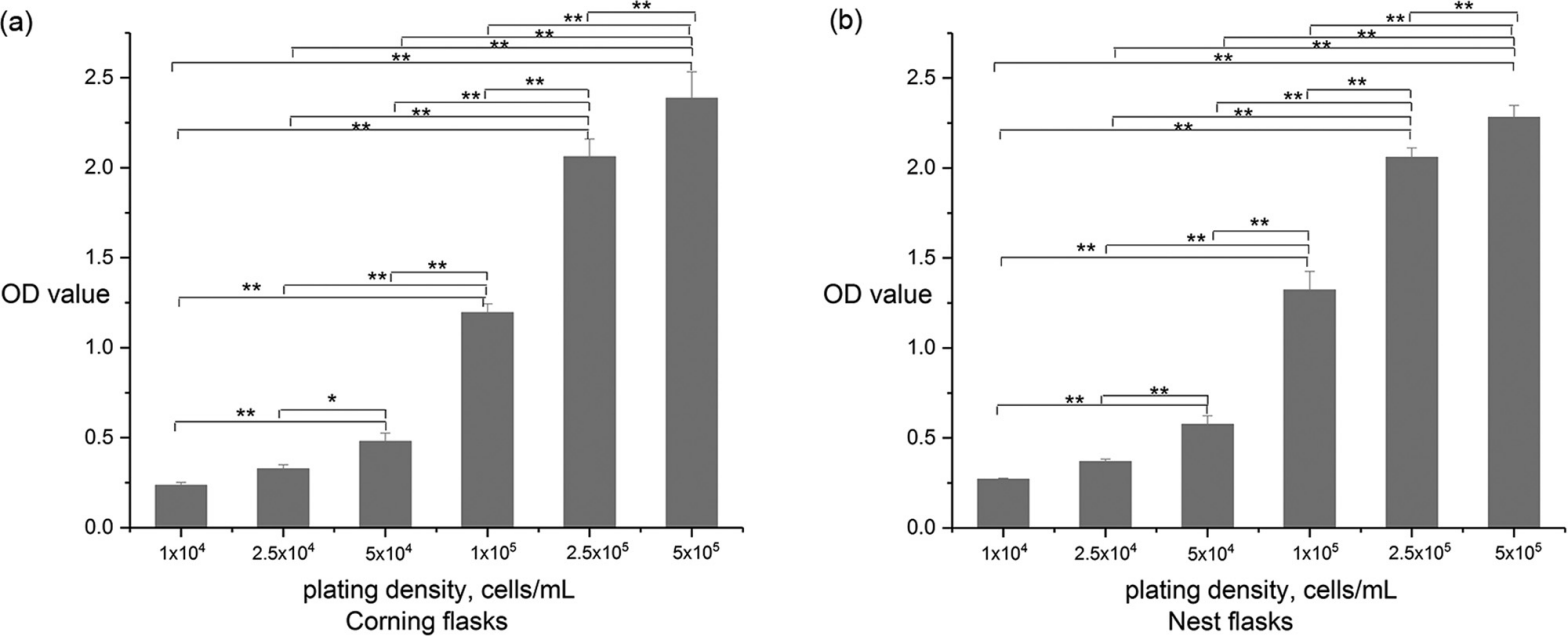
CCK8 results: the plating densities of 2.5 × 10^5^ and 5 × 10^5^ cells/mL promoted mSKP viability in both (a) Corning tissue-culture-treated flasks and (b) Nest untreated flasks (Results of three independent experiments, ANOVA for multiple comparisons, *p < 0.05, **p < 0.01.).

The CCK8 results indicated that compared with lower plating densities (10^4^–10^5^ cells/mL), the plating densities of 2.5 × 10^5^ cells/mL and 5 × 10^5^ cells/mL significantly promoted mSKP viability (*p* < 0.01) (Figure 3).

**Figure 4:**
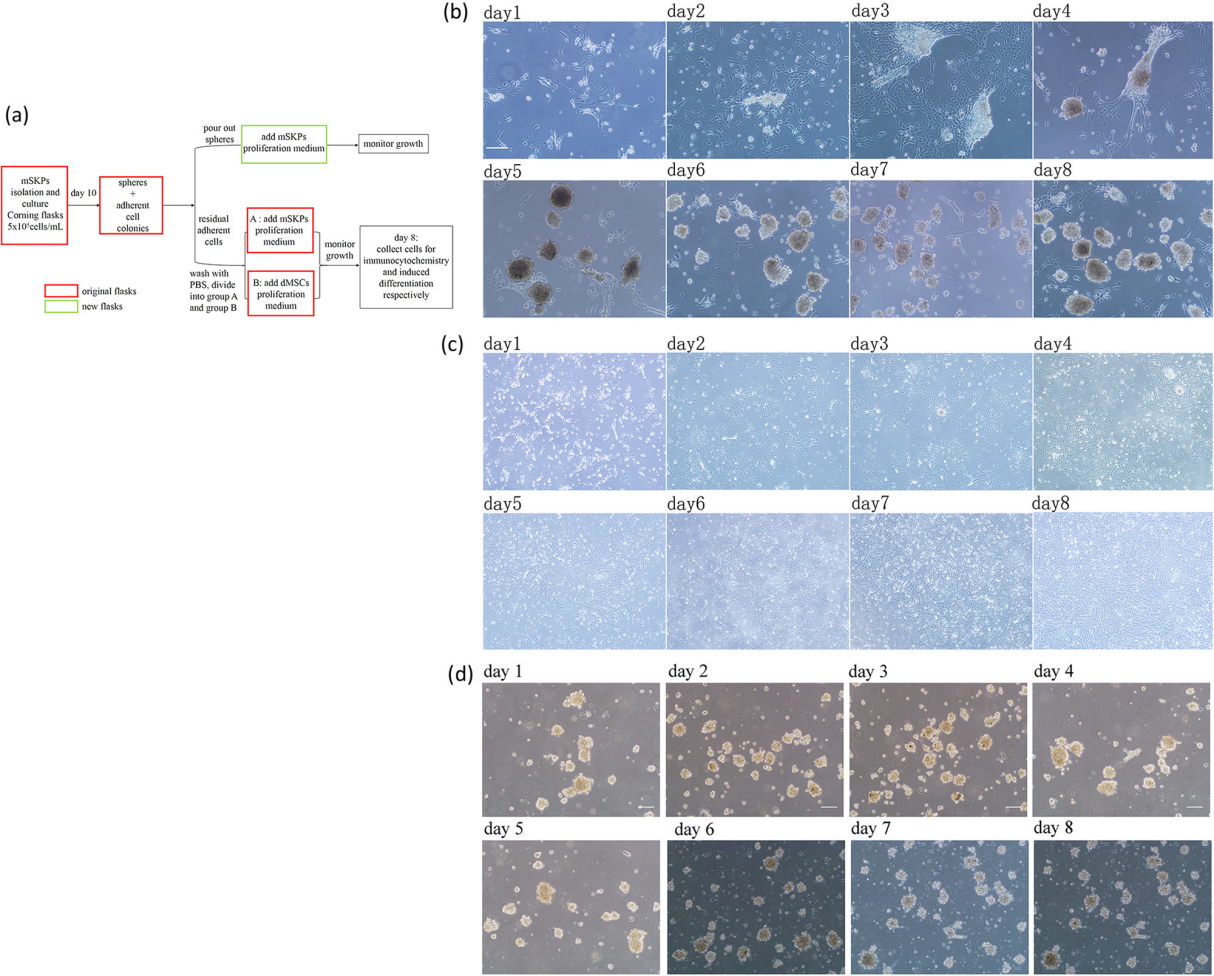
The experiment design and results of treating adherent cell colonies with culture condition A (group A) or B (group B). (a) The experiment design; (b) adherent cell colonies differentiated into SKP-like spheres under the SKP culture condition, in culture condition A, day 1–day 8; (c) adherent cell colonies differentiated into DMSC-like colonies under the DMSC culture condition, in culture condition B, day 1–day 8; (d) the poured-out spheres stopped growing in new flasks. Bar: 100 µm.

### 3.4 Treating adherent cell colonies with culture condition A or B

#### 3.4.1 Cell colonies in culture condition A or B

Culture condition A: on the 1st day, only a few residual adherent cells were noticed, and then the cells proliferated and formed multiple adherent cell colonies. On the 3rd or 4th day, spheres started to form on the colonies, attached and then detached and migrated from the adherent cell colonies as growing larger. On the 7th–8th day, a large number of spheres were noticed, while the adherent cell colonies reduced considerably (Figure 4b). Culture condition B: the residual adherent cells proliferated to form colonies. The cells demonstrated a flattened and fibroblast-like morphology, with a bright lining (Figure 4c).

#### 3.4.2 The poured-out spheres

Although being cultured in SKP medium and fed every 3 days with an addition of fresh medium containing all growth factors and supplements, the poured-out spheres stopped growing in both number and diameter in the new flasks (Figure 4d).

### 3.5 Antigen expression

**Figure 5:**
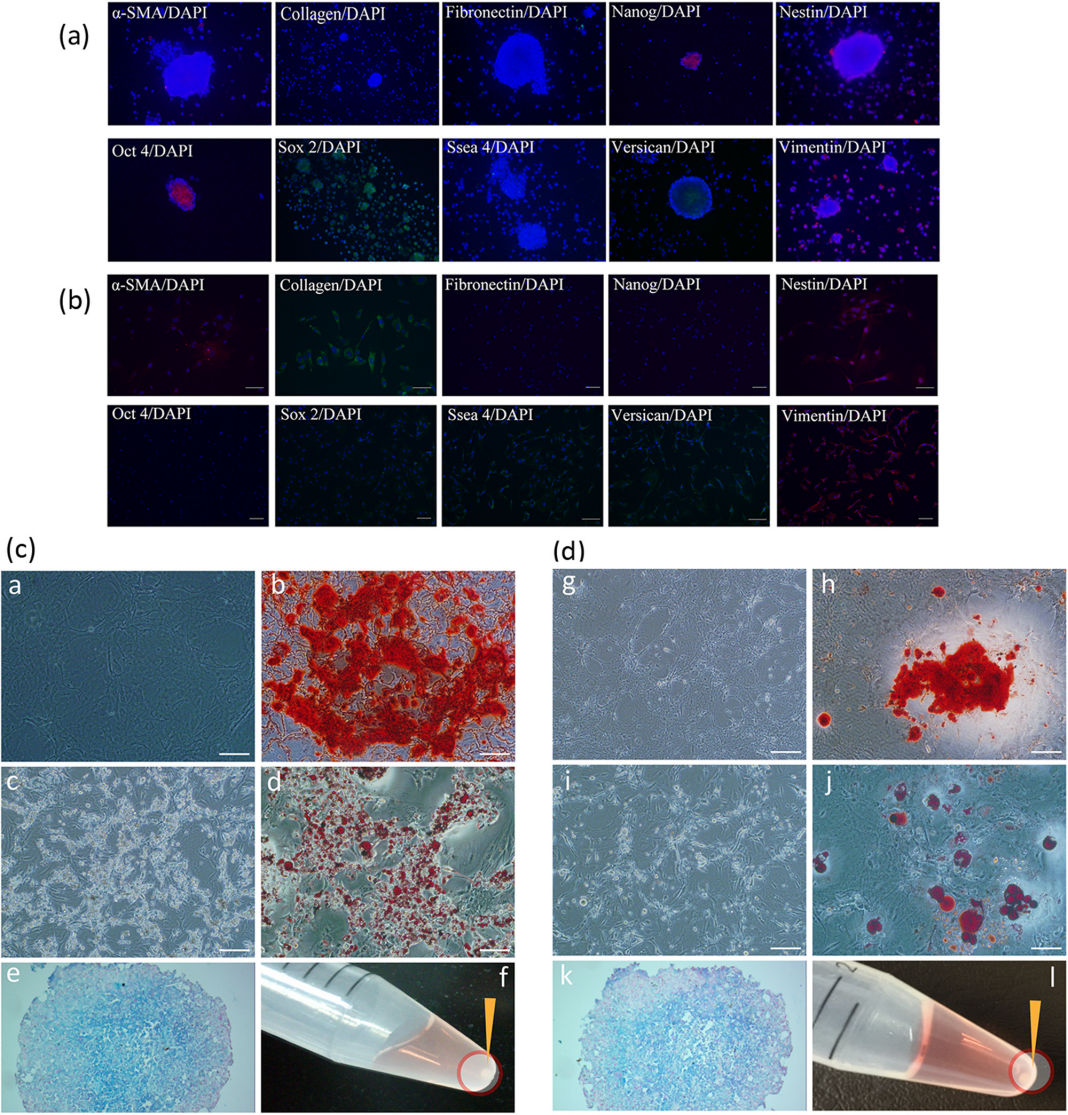
Results of immunocytochemistry and induced differentiation of cells obtained from culture condition A or B. (a) The spheres obtained from culture condition A expressed α-SMA, Nanog, Nestin, Oct4, Sox2, Ssea4, Versican, and Vimentin; (b) the adherent cells obtained from culture condition B expressed α-SMA, Collagen I, Nestin, Sox2 (weak positive), Ssea4, Versican, and Vimentin; (c) osteocyte-, adipocyte-, and chondrocyte-induced differentiation of spheres obtained from culture condition A; (d) osteocyte-, adipocyte-, and chondrocyte-induced differentiation of adherent cell colonies obtained from culture condition B. ((a and g) Osteocyte calcified matrix (osteogenesis, day 28); (b and h) alizarin red stain (day 28); (c and i) adipocyte lipid droplets (adipogenesis, day 28); (d and j) oil red stain (day 28); (e and k) alcian blue stain, pellets consisted of chondrocytes glycosaminoglycan (chondrogenesis, day 30); (f and l) chondrogenic pellets formed at the bottom of the tubes (indicated by arrowheads, day 30)). Bar: 100 µm.

According to the immunocytochemistry results, the spheres obtained from culture condition A expressed α-SMA, Nanog, Nestin, Oct4, Sox2, Ssea4, Versican, and Vimentin (Figure 5a). The adherent cells obtained from culture condition B expressed α-SMA, Collagen I, Nestin, Sox2 (weakly positive), Ssea4, Versican, and Vimentin (Figure 5b).

### 3.6 Induced differentiation

After a 28 day induction, cells obtained from both culture conditions A and B differentiated into osteocyte calcified matrix (osteogenesis) or adipocyte lipid droplets (adipogenesis) (Figure 5c and d). Chondrogenic pellets formed at the bottom of the 15 mL tubes after a 30 day induction slides stained with Alcian blue indicated pellets consisted of chondrocytes glycosaminoglycan (chondrogenesis) (Figure 5c and d).

## 4 Discussion

Biernaskie et al. [11] believed the growth rate of mSKPs was cell density dependent and recommended the optimal 10^4^–2.5 × 10^4^ cells/mL plating density for mSKPs. They noticed the adherent cell colonies under the serum-free culture condition, and they stated, “it is extremely important to use tissue-culture-treated plastic in the flasks to prevent adherence of cells to the plastic.” Nevertheless, according to our experience, mSKP growth remained very limited even if all the requirements mentioned above were fulfilled.

In this study, two types of flasks, including tissue-culture-treated flasks and untreated flasks, were used. For the tissue-culture-treated flasks, the cell culture surface treatment promotes cell attachment and growth. Untreated flasks are preferred in traditional SKP culture because SKPs are floating spheres, and the attachment of cells to the flask surface is unexpected. Noteworthily, our research revealed that surface treatment did not influence SKP growth. In addition, we explored the impact of plating density on mSKP production, as well as the biological characteristics of the adherent cell colonies. The research demonstrated that both mSKPs and adherent cell colonies increased as the plating density ascended. Inconsistent with Biernaskie’s findings [11], mSKPs formed on the adherent cell colonies firstly and then detached to form floating spheres as the amount and size increased. Lower plating densities (10^4^–10^5^ cells/mL) generated significantly less adherent cell colonies, which resulted in significantly fewer floating mSKP spheres. Taken together, the optimal plating density promoted mSKP proliferation by promoting adherent cell colonies growth. Conventionally adherent cells were avoided in SKP culture (for example, by decreasing cell density), which strictly limited SKP sphere development.

Considering mSKP spheres were 3D and might vary largely in size, we detected the spheres’ number, diameter, and cell viability when evaluating efficiency. All three parameters increased significantly under the optimal density. In addition, we managed to shorten the culture duration by 7 days by obtaining adequate mSKPs on the 12th or 14th day. More importantly, no extra growth factors, cytokines, additional apparatus, or biomedical materials would be needed, hence managed budget control. Optimizing plating density was easy to perform, and the SKP biological pattern would not be interrupted.

Vast chunks of mSKP aggregation were noticed on the 8th or 10th day at 106 cells/mL density. The dark center of aggregation implied insufficient nutrients and unhealthy cells, which implied that the plating density of 10^6^ cells/mL did not prove to be more productive than 5 × 10^5^ cells/mL. A similar situation was also noticed in hSKPs (data on file), and the plating density of 5 × 10^5^ cells/mL proved to be more productive than the lower densities (1 × 10^5^ and 2.5 × 10^5^ cells/mL).

Treating adherent cell colonies with two culture conditions further elucidated the correlation between mSKP spheres and adherent cell colonies. The cells that adhered to both tissue-culture-treated flasks and untreated flasks under a serum-free culture condition seemed to possess the capacity to proliferate and differentiate toward two distinct directions under two different culture conditions. The immunocytochemistry and induced differentiation results confirmed that the spheres achieved from culture condition A were mSKPs. The adherent cell colonies achieved from culture condition B displayed the most important markers and characteristics that a primary culture should possess to be classified as DMSCs [24]. Forni et al. [25] reported simultaneously isolating and characterizing three stem cell populations from the dermis and epidermis of murine skin, namely epidermal stem cells, SKPs, and DMSCs. They believed DMSCs and SKPs could be isolated simultaneously from one skin sample and showed no overt difference with DMSCs and SKPs isolated in a routine way. Nevertheless, we did not consider that multiple types of cells were isolated simultaneously. We believed that mSKPs and DMSCs are derived from the same cell type isolated (i.e., the adherent cell colonies), and different culture conditions generated two types of cells with distinct biological properties. Taken together, the characteristics of cells were shown to be altered by culture conditions.

Notably, the poured-out mSKP spheres failed to grow in new flasks even if cultures were fed with the addition of fresh medium containing all growth factors and supplements, implying mSKP spheres did not proliferate without the presence of adherent cell colonies. This finding confirmed again that mSKP spheres originated from the adherent cell colonies. It also explained the observation by Zong et al. [26], who reported isolating SKPs and DMSCs simultaneously from a single human skin sample. According to them, the suspended cells were collected 4 h later and transferred to another flask, and cultured in SKP proliferation medium. No obvious floating sphere formed even 10 days later, and they mentioned: “the definite reasons were unknown.” Interestingly, they also reported that many cells adhered to the base of the flask 4 h after isolation, which was not observed in our experiment. At the plating density of 5 × 10^5^ cells/mL, the adherent cells did not present until the 2nd or 3rd day.

In summary, mSKPs are derived from the adherent cell colonies in the primary cell culture, regardless of their floating characteristic. Within a certain limit, the plating density played a major role in mSKP development. The optimal plating density (2.5 × 10^5^–5 × 10^5^ cells/mL) accelerated mSKP growth and guaranteed the following stem cell-conditioned medium and exomes research. In addition, the research data on mouse stem cells might provide insights for hSKP culture and promote hSKP basic research and clinical application.
